# Stresses as First-Line Tools for Enhancing Lipid and Carotenoid Production in Microalgae

**DOI:** 10.3389/fbioe.2020.00610

**Published:** 2020-07-23

**Authors:** Tian-Qiong Shi, Ling-Ru Wang, Zi-Xu Zhang, Xiao-Man Sun, He Huang

**Affiliations:** School of Food Science and Pharmaceutical Engineering, Nanjing Normal University, Nanjing, China

**Keywords:** microalgae, stresses, lipids, carotenoids, omics technologies

## Abstract

Microalgae can produce high-value-added products such as lipids and carotenoids using light or sugars, and their biosynthesis mechanism can be triggered by various stress conditions. Under nutrient deprivation or environmental stresses, microalgal cells accumulate lipids as an energy-rich carbon storage battery and generate additional amounts of carotenoids to alleviate the oxidative damage induced by stress conditions. Though stressful conditions are unfavorable for biomass accumulation and can induce oxidative damage, stress-based strategies are widely used in this field due to their effectiveness and economy. For the overproduction of different target products, it is required and meaningful to deeply understand the effects and mechanisms of various stress conditions so as to provide guidance on choosing the appropriate stress conditions. Moreover, the underlying molecular mechanisms under stress conditions can be clarified by omics technologies, which exhibit enormous potential in guiding rational genetic engineering for improving lipid and carotenoid biosynthesis.

## Graphical Abstract

Development of new production microalgae strains with high lipid production capacity and cell growth with the aids of gene editing tools for selecting key genes under stress conditions.

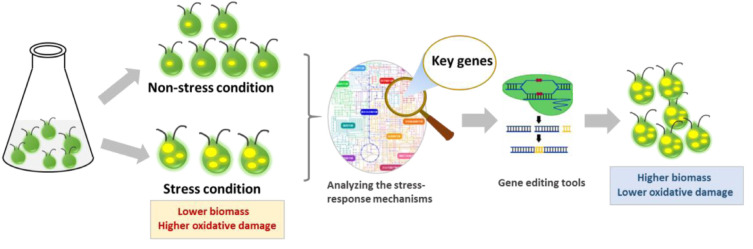

## Introduction

Microalgae have been considered as an alternative source of lipids and carotenoids (Chisti, 2007), the accumulations of which can usually be induced by stress conditions (Pribyl et al., 2012). Under extreme environmental conditions, microalgae synthesize and produce various secondary metabolites to retain their growth rate. In general, lipids act as an energy-rich carbon storage battery that enables the cells to survive under transient harsh environmental conditions (Hellier et al., 2015). A number of studies showed that stress conditions increased levels of intracellular reactive oxygen species (ROS), which in turn changed the carbon metabolic flux from glycolysis to the oxidative pentose phosphate pathway and subsequently resulted in the excessive accumulation of intracellular equivalents of excessive reduction, such as NADPH (Hu et al., 2008; Shi et al., 2017). In order to protect microalgae cells from the excessive reduction equivalents produced under stress, lipid, rather than protein or carbohydrate, was accumulated in large quantities due to the greater demands of NADPH. However, high levels of ROS can also cause damage to biological macromolecules such as DNA, lipids, and proteins (Ruenwai et al., 2011). To counter this danger, microalgae synthesize additional amounts of carotenoids to scavenge ROS under stress conditions.

Carotenoids can form a protective layer to prevent reactive radicals and lipid peroxidation. Moreover, carotenoids are important for maintaining the functionality of the photosynthetic apparatus and the integrity of membranes, which is essential for cell survival. Carotenoids can be divided into primary and secondary classes. Primary carotenoids are growth coupled metabolites located in the mitochondria membranes and chloroplast that suffer degradation under stress. Among the primary carotenoids, lutein is particularly prominent since it acts to maintain the membrane integrity and protects cells under many stress conditions (Sanchez et al., 2008). By contrast, secondary carotenoids can be produced under stress conditions. Astaxanthin is considered a typical secondary carotenoid and is present in cytoplasmic lipid bodies outside the chloroplasts (Lemoine and Schoefs, 2010). Moreover, primary carotenoids such as lutein are degraded under stress, while secondary carotenoids remain largely unaffected (Del Campo et al., 2000). However, several primary carotenoids, such as β-carotene, also accumulate under stress conditions and act as secondary metabolites (Hermawan et al., 2018).

**FIGURE 1:**
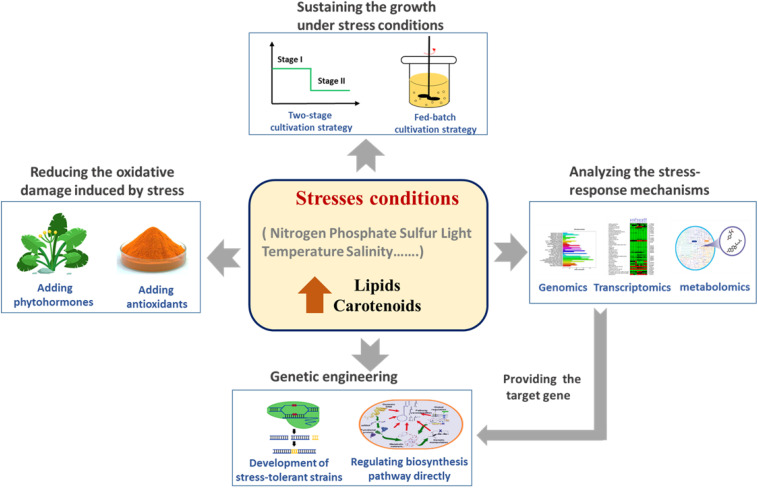
Stress conditions act as the triggers of lipid and carotenoid production in microalgae.

Many of strategies have been developed for overcoming the limitations of growth inhibition and oxidative damage, including two-stage cultivation strategies and the addition of growth-promoting agents (Figure 1). In fact, there are many inseparable links between the production of lipids and of carotenoids. Firstly, their biosynthesis pathways share the same precursor substrate, acetyl-CoA. Secondly, scavenging ROS from lipid peroxidation is an important characteristic of carotenoids. Lastly, carotenoids are toxic to the cell, so lipid droplets can act as “storerooms” for carotenoids (Ma et al., 2018). In this review, we summarize recent studies on stress-based fermentation strategies for the induction of lipid and carotenoid production in microalgae, including nutrient-related stresses and environmental stresses (Table 1). The underlying molecular mechanisms of the behavior of specific strains under stress conditions can increasingly also be clarified using omics technologies, which can guide rational genetic engineering for improving the biosynthesis of lipids and carotenoids.

**TABLE 1:** Effect of various stresses on the lipid and carotenoid production of microalgae.

Microalgae	Stress condition	Performance	References
*C. vulgaris*	Nitrogen limitation	Lipid content (% biomass) was increased from 30 to 89%	Shen et al. (2020)
*N. oleoabundans*	Nitrogen limitation	Lipid yield was increased 2-fold	Li et al. (2008)
*C. vulgaris*	Nitrogen limitation	Lipid productivity was increased from 226.5 to 305.7 mg L^–1^ day^–1^	Liu T. et al. (2016)
*C. zofingiensis*	Nitrogen limitation	Carotenoid content was increased from 0.05 to 4.5 mg g^–1^	Mulders et al. (2014)
*D. salina*	Nitrogen limitation	β-carotene productivity was increased from 1.28 to a maximum of 14.4 g LCV^–1^ day^–1^	Lamers et al. (2012)
*Scenedesmus* sp.	Phosphorus limitation	Lipid content was increased from 30% to 53%	Li X. et al. (2010)
*B. braunii*	Phosphorus limitation	Lipid yield was increased from 218 to 416 g L^–1^	Shen et al. (2020)
*C. regularis*	Sufficient phosphorus and mitrogen limitation	Lipid content was increased from 24.3% (50 mg L^–1^ nitrogen + 45 mg L^–1^ phosphorus) to 42.3% (250 mg L^–1^ nitrogen + 5.5 mg L^–1^ phosphorus)	Fu et al. (2017)
*C. vulagris*	Sulfur limitation	The maximum total lipid content of 53.43 ± 3.93% g gDW^–1^ was obtained	Sakarika and Kornaros (2017)
*P. tricornutum*	High light	TAG productivity was increased from 15.58 to 31.39 mg L^–1^ day^–1^	He et al. (2015)
*C. protothecoides*	High light	Total lipid content increased from 24.8 to 37.5%, and C16-18 fatty acids increased from 76.97 to 90.24%	Kurpan Nogueira et al. (2015)
*Desmodesmus* sp.	High light	A maximum lutein productivity of 3.6 mg L^–1^ day^–1^ was obtained	Lamers et al. (2010)
*D. salina*	High light	The highest β-carotene production of 30 pg cell^–1^ day^–1^ was obtained	Xie et al. (2013)
*H.pluvialis*	Nitrogen limitation and high light	Lipid and astaxanthin production reached 40.69 and 1.85%, respectively	Zhao et al. (2018)
*A. mangrovei*	Lower temperature	The DHA fraction was increased from 29 to 42%	Kanokwan and Cornelis (2012)
*N.salina*	Lower temperature	EPA content was increased 2- to 3-fold.	Guedes et al. (2010)
*Muriellopsis* sp.	High temperatures	A maximum lutein content of 0.54% d.wt. at 44°C was acquired	Del Campo et al. (2000)
*C. p*rotothecoides	High salinity	The highest lipid content of 41.2% and lipid yield of 185.8 mg g^–1^ were obtained under 30g L^–1^ NaCl condition	Wang et al. (2016)
*C. vulgaris*	High salinity	The lipid content was enhanced from 60 to 70% when the NaCl concentration was increased from 0.5 to 1.0 M	Takagi et al. (2006)
*H. pluvialis*	High salinity	The maximum astaxanthin content of 5.01 mg g^–1^ was obtained	Ding et al. (2019)
*D. salina*	High salinity	Resulted in a 30-fold increase in β-carotene production	Coesel et al. (2008)

## The Effects of Nutrient-Related Stresses and Generalized Stress-Response Mechanisms

### Nitrogen Limitation

#### Performance

A linear relationship between the concentration of the nitrogen source and lipid content was observed in many microalgal species (Hsieh and Wu, 2009). For example, lipid production increased 93% in *Chlamydomonas reinhardtii*, and *Acutodesmus dimorphus* accumulated 75% neutral lipids among total lipids under nitrogen-deficient conditions (Chokshi et al., 2017; Yang et al., 2018). It is possible that nitrogen-deficient conditions might improve microalgal lipid biosynthesis by affecting other biochemical pathways (Srinuanpan et al., 2018). The production of carotenoids, including β-carotene, astaxanthin, and lutein, was also successfully enhanced by nitrogen limitation in *Chlorella zofingiensis*, *Dunaliella salina, Neochloris oleoabundans*, and *Muriellopsis* sp. (Del Campo et al., 2000; Mulders et al., 2014; Urreta et al., 2014). Nitrogen deprivation can also promote the concomitant accumulation of both lipids and the antioxidant pigment astaxanthin in microalgae (Chen et al., 2015; Liu J. et al., 2016).

#### Possible Mechanisms

On the one hand, low nitrogen levels reduce competition for carbon, which is required for the synthesis of both carotenoids and proteins, since only the latter require nitrogen (Wang et al., 2019). Another possible reason might be the degradation of nitrogenous compounds such as chlorophyll and proteins, which might provide carbon or energy for the accumulation of lipids (Liu T. et al., 2016). Moreover, it has been reported that nitrogen deficiency can upregulate diacylglycerol acyltransferase (DGAT), which catalyzes the last step of TAG assembly in many microalgal species (Li-Beisson et al., 2015). Overall, these adaptive responses help to ensure the survival of cells during times of stress. While lipids serve as energy stores, astaxanthin seems to play a role in the protection against ROS (Rosenberg et al., 2008).

### Phosphate Limitation

#### Performance

Phosphate limitation also has a positive effect on the accumulation of lipids or carotenoids (Li X. et al., 2010; Shen et al., 2020). Sun et al. (2014) found that phosphate limitation shortened the fermentation time, leading to the highest DHA productivity of 291 mg L^–1^ h^–1^ in *Schizochytrium* sp. Interestingly, Chu et al. (2014) found that a combination of sufficient phosphorus and nitrogen starvation was the real “lipid trigger.” It has been reported that expressions of enzymes involved in glycolysis and pentose phosphate pathway were enhanced greatly under nitrogen limitation (Rai et al., 2017), which indicated that more energy was needed in this process. Inorganic phosphate is important to cellular energy transduction (Beardall et al., 2005). Based on this, sufficient phosphorus can improve biomass productivity in the nitrogen starvation stage, which has been shown in *Chlorella* sp. and *Scenedesmus obliquus* (Fu et al., 2017; Shen et al., 2020). Therefore, regulating lipid production through a combination of sufficient phosphorus and nitrogen starvation seems to be an economical and environmentally friendly approach.

#### Possible Mechanisms

Phosphate can influence carbon flow between the starch and lipid synthesis pathways by regulating the activity of ADP-glucose pyrophosphorylase (Yao et al., 2018), which is the first committed step of starch synthesis. Since starch biosynthesis shares common carbon precursors with lipid biosynthesis, blocking the starch biosynthesis pathway can increase the metabolic flux toward lipid accumulation. Inhibition of ADP-glucose pyrophosphorylase in *Chlamydomonas* resulted in a 10-fold increase of lipid yield (Li Y. et al., 2010).

### Sulfur Limitation

#### Performance

Sulfur limitation is also beneficial for lipid production in microalgae. For example, sulfur starvation was found to be more effective for increasing TAG production in *C. reinhardtii* than was nitrogen and phosphorus depletion (Sakarika and Kornaros, 2017). It has been reported that starvation of sulfur is more efficient than that of phosphorus or iron for inducing high-level accumulation of astaxanthin (Boussiba and Vonshak, 1991). However, phosphorus starvation led to the highest increase of β-carotene accumulation in another report (Phadwal and Singh, 2003). Therefore, the effect of sulfur limitation on lipids and carotenoids depends on the microalgae species.

#### Possible Mechanisms

Sulfur is necessary for the synthesis of the key antioxidant molecule glutathione (GSH; Carfagna et al., 2016), which participates in the stress response by scavenging ROS. Therefore, an increase of ROS caused by sulfur limitation and a consequent decrease of intracellular GSH concentration can enhance the production of carotenoids as alternative ROS scavengers. Additionally, many authors suggested that GSH might act as a “sensor” of the cell’s sulfur status, thus regulating the rate of sulfur assimilation (Carmen Ruiz-Dominguez et al., 2015; Carfagna et al., 2016).

## The Effects and Generalized Mechanisms of Environmental Stresses

### Light Stress

#### Performance

Under high light intensity, the total polar lipid content decreased, while the contents of neutral storage lipids increased (Hu et al., 2008). Based on these findings, high light intensity was used to trigger the accumulation of neutral lipids in *Phaeodactylum tricornutum, Chlorella* sp., and *Chlorella protothecoides* (He et al., 2015; Krzeminska et al., 2015; Kurpan Nogueira et al., 2015). Light stress has often been applied in conjunction with nitrogen (Ho et al., 2015; Remmers et al., 2017), salinity (Pal et al., 2011), and temperature stress (Kurpan Nogueira et al., 2015). Zhao et al. (2018) showed that a combination of nitrogen limitation and high light stress simultaneously increased the accumulation of lipids and astaxanthin in *Haematococcus pluvialis*, which reached 40.69 and 1.85% of the cell dry weight, respectively. Under high light intensity, maximum lutein productivity (3.6 mg L^–1^ day^–1^) and β-carotene productivity (30 pg cell^–1^ day^–1^) were obtained in *Desmodesmus* sp. and *D. salina*, respectively (Lamers et al., 2010; Xie et al., 2013).

#### Possible Mechanisms

Directly, high light intensity promotes the cell growth of microalgae by enhancing photosynthesis. Some studies indicated that increased light intensity resulted in an increase in superoxide and hydrogen peroxide, which induced oxidative damage to polyunsaturated fatty acids (PUFAs), which finally resulted in a higher neutral lipid content and lower polar lipid content (He et al., 2015). Conversely, low light conditions are often reported as a factor for increasing the PUFA contents, which might be due to an increase of the amount of thylakoid membranes to counterbalance the lower availability of light (Remmers et al., 2017). High light intensity can boost the expression levels of the lycopene beta-cyclase gene (Ramos et al., 2008), which is the key enzyme for carotenoid accumulation in microalgae. Moreover, Coesel et al. (2008) also suggested that high light stress can regulate the activity of phytoene synthase and phytoene desaturase.

### Temperature Stress

#### Performance

It has been shown that many microalgae display increased growth and total lipid production at higher temperatures (Sayegh and Montagnes, 2011). Examples include *Nannochloropsis salina* and *Ettlia oleoabundans* (Converti et al., 2009; Yang et al., 2013). In fact, temperature stress has a greater effect on the lipid composition than on total lipid yield, especially for PUFA biosynthesis (Guedes et al., 2010; Van Wagenen et al., 2012). However, high temperatures are beneficial for lutein accumulation in microalgae such as *Muriellopsis* sp. and *Scenedesmus almeriensis* (Del Campo et al., 2000; Sanchez et al., 2008). By contrast, slightly low temperature during the night period was beneficial for both growth and astaxanthin accumulation in *H. pluvialis* (Wan et al., 2014). Thus, the optimum culture conditions are different in different microalgae species because of the localized sensitivity of microalgae to nutrients, and the optimal nutrient concentrations for one strain may inhibit the growth of other strains.

#### Possible Mechanisms

At lower temperature, more PUFAs are synthesized and incorporated into the membrane to maintain its fluidity. Moreover, Ward and Singh (2005), as well as An et al. (2013), view the higher proportion of unsaturated fatty acids at lower temperatures as mainly a consequence of the higher dissolved oxygen concentration under these conditions, which directly stimulates the function of oxygen-dependent desaturases. It has been reported that higher temperatures result in increased photooxidative stress, which is correlated with increased carotenoid production in microalgal species (Tripathi et al., 2002). Low temperature decreases the nutrient uptake rate and slows lutein accumulation (Bhosale, 2004).

### Salinity Stress

#### Performance

The stimulating effect of increased salinity on lipid accumulation can be attributed to osmotic stress in terms of cellular responses (Pugkaew et al., 2019). Lipid accumulation in *Chlorella vulgaris* and *Dunaliella* sp. was greatly increased under high salinity stress, reaching 21.1 and 70%, respectively (Takagi et al., 2006). Interestingly, salt stress can also have a positive effect on the production of astaxanthin and β-carotene. For instance, the maximum astaxanthin content of 5.01 mg g^–1^ was obtained in *H. pluvialis* when treated with 0.2 g L^–1^ NaCl, which was 42% higher than that of the control (Ding et al., 2019). A general enhancement of astaxanthin production was also observed in *C. zofingiensis* at NaCl concentrations of up to 0.2 M (Campo et al., 2004). A moderate increase in salt concentration from 4 to 9% resulted in a 30-fold increase of β-carotene accumulation in *D. salina* (Coesel et al., 2008). However, salinity stress did not affect the content of lutein (Sanchez et al., 2008).

#### Possible Mechanisms

Salinity stress was regarded as a powerful regulator for switching the carbon distribution from starch to lipids in microalgae (Ho et al., 2017). Under high salinity stress, lipid production was increased, while the protein and starch content decreased. It was founded that ACCase (the key enzyme of lipid synthesis) was upregulated and AGPase (the key enzyme of starch synthesis) was downregulated. Moreover, the mRNA levels of key enzymes involved in starch degradation were greatly upregulated (Ho et al., 2017), which further proved that salinity induced the conversion of starch to lipids. In addition, salinity stress induced ROS generation. It has been proved that ROS can trigger lipid overproduction via several pathways. For example, ROS can enhance the activity of ACCase enzyme and regulate cellular autophagy (Goodson et al., 2011).

## Application of “omics” Technologies

The stress-response mechanisms of different microalgal species sometimes differ greatly. Although these systems are complex and involve a great number of genes, “omics” approaches, such as genomics, transcriptomics, proteomics, metabolomics, and lipidomics, offer powerful tools for examining the differential expression or regulation of genes under stress conditions (Guarnieri and Pienkos, 2015). It will be important to understand the key factors responsible for the overproduction of lipids, since this knowledge can be used to identify target genes for genetic engineering.

### Unraveling the Stress-Response Mechanisms of Microalgae

As the most commonly utilized method for lipid production, it is critical to investigate relevant metabolic regulation mechanisms under nitrogen limitation. For example, in *P. tricornutum*, a label-free quantitative proteomics approach revealed that nitrogen starvation significantly upregulated the expression of the β-subunit of methylcrotonyl-CoA carboxylase (MCC2) and that inhibition of MCC2 expression resulted in decreased lipid accumulation (Ge et al., 2014). Metabolomic analysis revealed that the PSR1 gene (phosphorus starvation response) is critical for lipid accumulation in response to phosphorus starvation (Bajhaiya et al., 2016). Recently, a lipidomic analysis of *Thalassiosira pseudonana* under phosphorus limitation found that the abundance of several diglycosylceramide lipids was increased up to 10-fold, revealing a novel class of substitute lipids and potential biomarkers for the study of phosphorus limitation (Hunter et al., 2018).

To obtain insights into the astaxanthin synthesis mechanism under high light conditions, Gwak et al. (2014) used transcriptome analysis and found that beta-carotene hydroxylase (BKT or crtZ), phytoene synthase (PSY), and phytoene desaturase (PDS) were all upregulated in *H. pluviali*s under high irradiance. Analogously, transcriptome analysis of *H. pluviali*s revealed that high light intensities impacted the expression of genes involved in the carotenoid biosynthesis pathway, including PDS, crtISO, LcyB, LUT1, LUT5, and ZEP (Gwak et al., 2014; He et al., 2018). When the crtO ketolase gene was overexpressed in *H. pluviali*s, the accumulation of astaxanthin and other ketocarotenoids was greatly increased (Kim and Portis, 2004).

Under cold stress, the cellular content of PUFAs can be significantly increased to maintain membrane fluidity and functions. Consequently, Ma et al. (2015) used Illumina’s sequencing technology to examine global changes in the transcriptome of *Aurantiochytrium* sp. in response to low-temperature stress. Furthermore, proteomics was used to interpret the molecular mechanisms underlying the increase of DHA contents of *Aurantiochytrium* sp. at low temperatures. The results showed that low temperatures inhibited cellular energy generation through glycolysis and the TCA cycle and led to a significant up-regulation of polyunsaturated fatty acid synthase (Ma et al., 2017).

### Theoretical Guidance for Rational Genetic Engineering

Genetic engineering of microalgae guided by omics technologies has been increasingly applied. Based on an analysis of the transcriptome and lipidome of *C. reinhardtii* under heat stress, the key rate-limiting enzyme of DGAT was identified (Légeret et al., 2016). Overexpression of DGAT in *C. reinhardtii* resulted to a 9-fold increase in TAG content (Lamers et al., 2010). Similarity, DGAT was overexpressed in *P. tricornutum*, and the lipid content was enhanced from 27.5 to 37.2% (Remmers et al., 2017). Similar positive effects were also obtained in *Nannochloropsis oceanica*, *S. obliquus*, and *Chlorella minutissima* (Chen et al., 2016; Wei et al., 2017).

In addition to specific genes, omics technologies can identify global transcription factors that control lipid biosynthesis. For example, the PSR1 gene is related to stress due to phosphorus limitation. When PSR1 was overexpressed in *C. reinhardtii*, TAG accumulation was increased (Ngan et al., 2015). Recently, the transcription factor lipid remodeling regulator 1 (LRL1) was found in *C. reinhardtii* by transcriptomic analysis, which is a regulator of both membrane remodeling and TAG biosynthesis under phosphorus-limitation (Hidayati et al., 2019). In the future, LRL1 could be a potential target of engineering for improving lipid accumulation in microalgae.

Most stress-based induction strategies in microalgae generate ROS as by-products (Zhao et al., 2019). Antioxidant enzymes act as the first line of cellular defense against oxidative damage. In *Schizochytrium* sp., the expression levels of superoxide dismutase (SOD) and catalase (CAT) under high salinity stress were upregulated by 4.82 and 3.11-fold, respectively. Similar effects were also observed in other microalgal species, which indicated that enhancing the activity of antioxidant enzymes may be beneficial for lipid production (Ugya et al., 2020). Accordingly, when SOD was overexpressed in *Schizochytrium* sp., the ROS levels were greatly reduced and the PUFA titer was increased by 32.9% (Zhang et al., 2018).

## Conclusion

Each species of microalgae has different optimal stress conditions for the overproduction of desired metabolites. In total, stress-based strategy is the most effective way to enhance lipid or carotenoid production in microalgae, although this might inhibit cell growth and induce oxidative damage. According to extensive analyses on technology and economy, a two-stage cultivation strategy gives rise to more operational and capital costs compared to a single cultivation system. Therefore, working out how to economically improve growth under stress conditions is a future development trend. Moreover, the key factors influencing cell growth and production of valuable molecules still need to be clarified. In recent years, massive “omics” datasets continue to be used to identify the key regulatory genes that are activated under stress conditions. However, our understanding of microalgal stress responses is currently limited to model species and prediction of gene function based on homology. The availability of genetic tools for microalgae is limited, and many are not well developed. Therefore, it is necessary to develop functional genomics research by applying multiple omics technologies and establish effective genetic manipulation tools for non-model microalgae species.

## Author Contributions

T-QS and L-RW conceived the idea and wrote the first draft of the manuscript. Z-XZ designed the figures and drafted the table. X-MS contributed to manuscript revision and approved the final version. All authors read and approved the final manuscript.

## Conflict of Interest

The authors declare that the research was conducted in the absence of any commercial or financial relationships that could be construed as a potential conflict of interest.
